# Clinical and imaging characterization of ocular toxoplasmosis in a Central-West Brazilian population

**DOI:** 10.1007/s10792-026-04302-5

**Published:** 2026-10-17

**Authors:** Alexsandra Passarelli Giabardo Marques, Luiza Roschel Zaunrith, Danilo Donizete da Silva, Aparecida Perpétuo Silveira Carvalho, Fernando Henrique Antunes Murata, Rubens Camargo Siqueira, Lucas Gazarini, Edis Belini Júnior, Julie Massayo Maeda Oda, Christiane Maria Ayo, Luiz Carlos de Mattos, Cinara Cássia Brandão

**Affiliations:** 1 Universidade Federal do Mato Grosso do Sul, Três Lagoas, MS Brazil; 2 Department of Dermatological, Infectious and Parasitic Diseases, Faculdade de Medicina de São José do Rio Preto, FAMERP Toxoplasma Research Group, Avenida Brigadeiro Faria Lima, 5416, São José do Rio Preto, SP 15090-000 Brazil

**Keywords:** *Toxoplasma gondii*, Ocular toxoplasmosis, Color fundus photography, Retinal mapping, Serology

## Abstract

**Objective:**

To evaluate and characterize patients with presumed ocular toxoplasmosis using combined clinical and serological approaches in the municipality of Três Lagoas, Mato Grosso do Sul, Brazil.

**Methods:**

This cross-sectional quantitative study included 85 patients with presumed *Toxoplasma gondii* related retinochoroiditis referred from the public healthcare system. Participants underwent ophthalmologic examination and serological testing for anti-*T. gondii* IgG and IgM antibodies. Patients were classified into control (G1), active lesion (G2), and cicatricial lesion (G3) groups.

**Results:**

Overall, 76.5% of patients were positive for anti-*T. gondii* IgG, while 3.5% were positive for IgM. Most patients with cicatricial lesions (G3) were IgG-positive (31/33). In contrast, IgM positivity was restricted to a small subset of patients (3/13) with active lesions (G2). Ophthalmologic evaluation demonstrated that unilateral disease predominated (78.2%), with lesions mainly located in the posterior pole (52.8%). Morphologically, round-to-ovoid lesions were most frequent (60.0%), followed by amoeboid (16.0%), placoid (8.0%), and wedge-shaped and punctate (4.0% each) patterns. Mean lesion size among evaluable eyes was 16.2 mm^2^.

**Conclusion:**

The analyses provide data that contribute to the understanding of the clinical characteristics of the disease, ocular behavior, and serological findings. Consequently, preventive measures may be implemented, reducing risk factors and improving patients’ quality of life.

## Introduction

Toxoplasmosis is a globally distributed zoonotic disease and represents a significant public health challenge, particularly in developing countries [1]. It is estimated that approximately one-third of the world’s population is infected with *Toxoplasma gondii* [1, 2]. Following infection, the parasite establishes a chronic, lifelong condition in the host, with clinical manifestations that may vary depending on several factors, including host and parasite characteristics [3].

Although most infected individuals remain asymptomatic, in some cases, toxoplasmosis can cause severe disease, particularly in cases of immunosuppression. In immunocompromised individuals, it may progress to potentially fatal encephalitis, and when acquired during pregnancy, it can result in miscarriage or congenital malformations in the fetus; and even in immunocompetent individuals, it may cause serious complications, such as significant ocular involvement [4].

Ocular toxoplasmosis is one of the most common infectious causes of posterior uveitis and typically presents as retinochoroiditis [5, 6]. Diagnosis is primarily based on the identification of characteristic clinical findings during ophthalmologic examination and may be supported by specific serological tests for *T. gondii* [7, 8]. Primary ocular toxoplasmosis, although not always clinically evident, should be considered in the differential diagnosis of retinal and choroidal inflammation, even in the absence of detectable antibodies, as delayed diagnosis may compromise therapeutic management and visual prognosis [5, 9, 10]. Toxoplasmic lesions can be classified according to their stage of evolution as active, resolving, or cicatricial [5, 10–12]. Active retinochoroiditis typically appears as a whitish-yellow or grayish retinal lesion with poorly defined margins and associated vitreous inflammation, whereas cicatricial lesions are characterized by well-demarcated areas of retinochoroidal atrophy with variable pigment accumulation along or within the lesion [13]. Retinal vessels may traverse active lesions and may show reactive sheathing or, in severe cases, occlusion. In cicatricial lesions, the underlying tissue loss may result in a whitish appearance related to scleral exposure. These clinical features are important for distinguishing active inflammatory disease from established retinal scars and for characterizing the clinical spectrum of ocular toxoplasmosis [6, 10, 11, 13]. These clinical features reflect the evolution from active inflammatory retinal damage to a residual atrophic and pigmented scar and provide an important basis for the clinical classification of ocular toxoplasmosis [10, 11]. The pathogenesis of ocular toxoplasmosis, particularly in recurrent cases, remains complex and not fully understood. Evidence suggests that *T. gondii* may reach the eye via hematogenous spread, either as free tachyzoites or transported within circulating leukocytes [14]. Additionally, dissemination from the central nervous system through the optic nerve has been proposed, which may explain the juxtapapillary localization observed in some cases [14].

In Brazil, ocular toxoplasmosis is a leading infectious cause of posterior uveitis and a significant, preventable cause of visual impairment. [11, 15–18] Despite its clinical importance, regional data from the Central-West region remain scarce, particularly regarding lesion morphology, anatomical distribution, and lesion size as measured by retinal imaging techniques.

Despite recent advances in diagnostic and therapeutic approaches, ocular toxoplasmosis remains an important cause of visual morbidity [5, 6]. Clinical manifestations vary according to lesion size, location, and degree of inflammation, potentially leading to significant visual impairment [9, 19]. In this context, understanding the clinical and epidemiological patterns of the disease, both globally and regionally, is essential for improving diagnostic protocols, preventive strategies, and control measures [20, 21]. Therefore, the present study aims to evaluate and characterize, through clinical and laboratory approaches, patients with suspected ocular toxoplasmosis treated in the municipality of Três Lagoas, Central-West Region of Brazil.

## Methodology

This is a cross-sectional, quantitative study based on the collection of primary data obtained through clinical ophthalmologic evaluation and laboratory analyses of patients treated within the public healthcare system of the municipality of Três Lagoas, Mato Grosso do Sul state, Central-West Region of Brazil. The study aimed to characterize the main clinical, morphological, and serological findings associated with ocular toxoplasmosis in the evaluated population.

All participants underwent a targeted anamnesis focused on ocular toxoplasmosis, followed by a comprehensive ophthalmologic examination. The evaluation included assessment of visual acuity, anterior segment biomicroscopy, and fundoscopic examination under pharmacological mydriasis. Patients were enrolled in the study between May 2022 and July 2023. All participants were immunocompetent and were not receiving corticosteroids or other immunosuppressive medications at the time of clinical evaluation. In addition, none was receiving specific anti-*Toxoplasma gondii* therapy at the time of ophthalmological examination, retinal imaging, or blood collection. Previous anti-*Toxoplasma gondii* treatment was recorded when applicable.

Retinal mapping was performed using indirect ophthalmoscopy with the Vantage Plus ophthalmoscope (Keeler, USA). Lesion documentation was obtained through color fundus photography using a portable retinal camera (Eyer, Phelcom Technologies, Brazil) [22], allowing detailed recording and morphological analysis of the identified retinochoroidal alterations. Subsequently, peripheral blood samples were collected for serological analysis, including determination of anti-*Toxoplasma gondii* IgG and IgM by ELISA (EUROIMMUN AG, Lübeck, Germany). Clinical and lesion-related variables were defined according to standardized clinical reporting principles proposed by the Standardization of Uveitis Nomenclature (SUN) Working Group [23]. Lesion morphology was categorized according to the geometric configuration as round or ovoid, amoeboid, placoid, wedge-shaped, or punctate. Lesion characteristics were assessed separately according to their distribution pattern and classified as circumferential or confluent. Anatomical location was evaluated independently according to retinal topography, including the posterior pole, mid-periphery, and peripheral retina. Thus, morphology, distribution pattern, and anatomical location were considered complementary descriptive domains for the characterization of each lesion.

### Serological assay validation and treatment status

Anti-*Toxoplasma gondii* IgG and IgM were determined using a commercially available ELISA assay (EUROIMMUN AG, Lübeck, Germany), according to the manufacturer’s instruction. Peripheral blood samples were collected at the time of the ophthalmologic evaluation, before initiation of specific anti-toxoplasmic therapy in treatment-naive patients at the laboratory of the Faculty of Medicine of São José do Rio Preto (FAMERP). Serological testing was performed independently of the clinical activity of the ocular lesion and was not scheduled according to lesion stage. The treatment status of each participant at the time of serological testing was recorded, including previous anti-*Toxoplasma gondii* treatment when applicable.

Assay performance as reported by the manufacturer is a sensitivity of > 95% and a specificity of >98% for IgG, with calibrators and positive and negative controls included in every run and results expressed in relative units per millilitre against the World Health Organization (WHO) international standard. Borderline results were retested; samples with discordant results between techniques were excluded, as stated in the exclusion criteria.

Based on ophthalmologic and laboratory findings, participants were classified into three groups: (G1) patients with non-toxoplasma ocular diseases, constituting the control group (n = 39); (G2) patients with ocular toxoplasmosis presenting active lesions (n = 13); and (G3) patients with ocular toxoplasmosis presenting cicatricial lesions (n = 33).

### Control group (G1)

The control group comprised patients referred to the same service and examined by the same protocol who had ocular disease unrelated to toxoplasmosis and no fundoscopic evidence of toxoplasmic retinochoroiditis. The diagnoses in this group were: diabetic retinopathy, age-related macular degeneration, glaucoma and glaucoma suspects, refractive errors with otherwise normal fundus, cataract, hypertensive retinopathy, non-toxoplasmic peripheral retinal degenerations, and other non-infectious retinal disorders. Controls were recruited consecutively from the same referral population and over the same period as cases, in order to minimise selection bias; nevertheless, because all participants were drawn from a tertiary referral service, residual referral bias cannot be excluded.

### Diagnostic criteria and classification

The diagnosis of presumed ocular toxoplasmosis was established on clinical grounds by a single experienced retina specialist, based on predefined fundoscopic criteria, and was made independently of, and prior to, the serological results. The diagnosis of presumed ocular toxoplasmosis was established on clinical grounds by a single experienced retina specialist, based on predefined fundoscopic criteria, and was made independently of, and prior to, the serological results. Active toxoplasmic retinochoroiditis was defined as a focal whitish-yellow or grayish retinal lesion with poorly defined borders accompanied by overlying vitreous inflammation, with or without an adjacent pigmented scar. Cicatricial (inactive) disease was defined as a well-demarcated chorioretinal atrophic lesion with variable pigment accumulation along or within the lesion, in the absence of vitreous inflammation or lesion border blurring [13]. The presence or absence of vitreous inflammation was assessed qualitatively as part of this clinical distinction; because no standardized vitreous inflammation grading scale had been incorporated into the original data-collection protocol, its severity was not quantitatively graded. These criteria were based on the characteristic clinical features of active and cicatricial toxoplasmic retinochoroiditis described in the literature [13].

Because anti-*Toxoplasma gondii* IgG seroprevalence is high in the Brazilian general population, serology alone was not used to establish the diagnosis; it was interpreted as supportive evidence only, in conjunction with the clinical phenotype. Alternative causes of retinochoroiditis were excluded on clinical and, when indicated, laboratory grounds, including syphilis, tuberculosis, sarcoidosis, viral retinitis (herpes simplex, varicella-zoster and cytomegalovirus), toxocariasis and non-infectious posterior uveitis. Patients with atypical lesion morphology or with clinical features suggesting an alternative aetiology were not classified as toxoplasmic and were not included in G2 or G3.

**Fig. 1:**
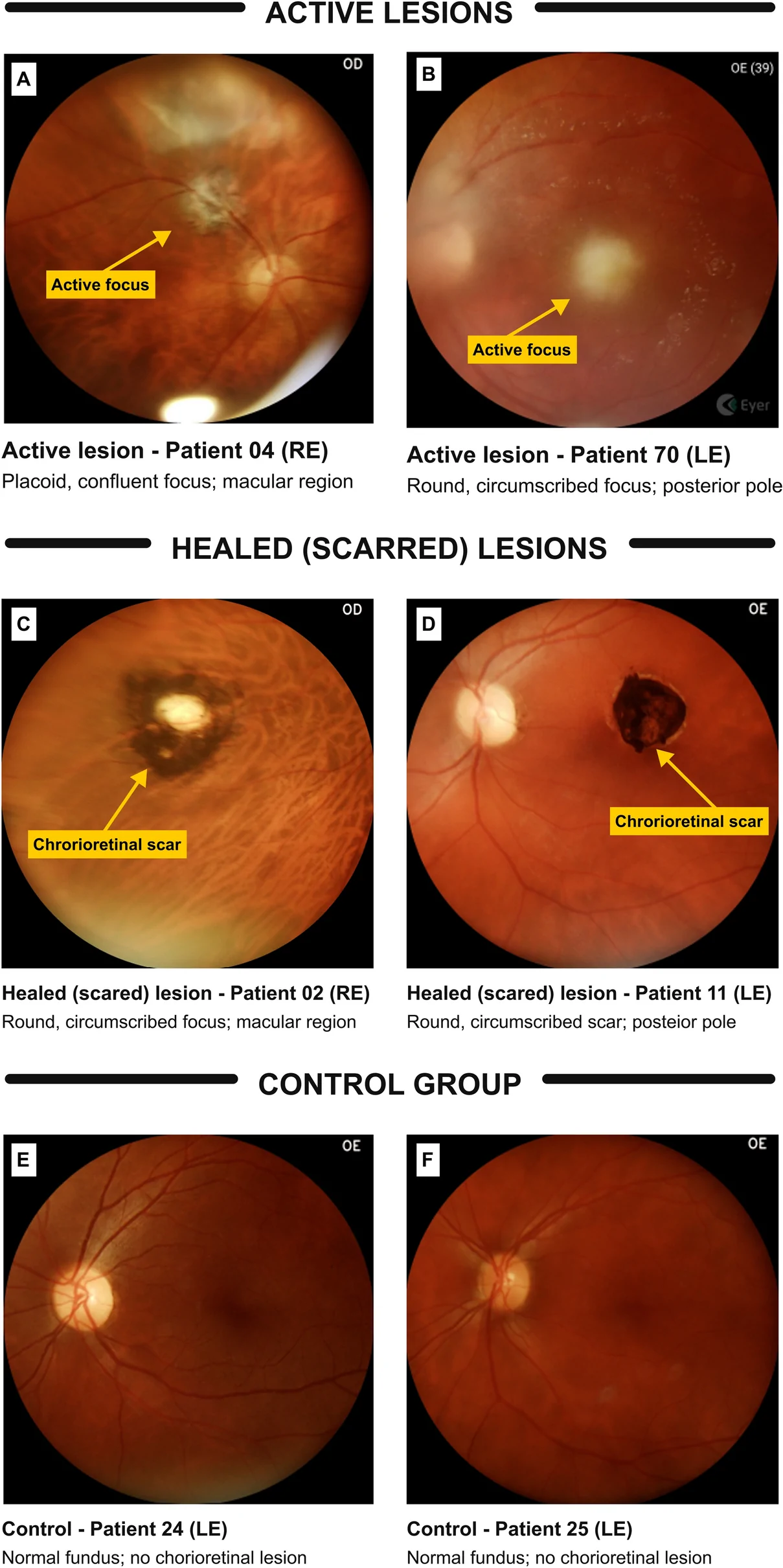
Comparative fundus retinography panel illustrating active and scarred lesions of presumed ocular toxoplasmosis alongside normal control fundi, using a portable retinal camera (Eyer, Phelcom Technologies, Brazil). Top row (**A**, **B**): Active retinochoroiditis lesions. **A** Patient 04, right eye (RE): a placoid, confluent, yellowish-white active focus adjacent to the optic disc involving the macular region, with indistinct, feathered borders and overlying vitreous haze, consistent with an active inflammatory reaction. **B** Patient 70, left eye (LE): a round, well-circumscribed, creamy-yellow active focus located in the posterior pole, exhibiting the typical satellite pattern adjacent to a pre-existing chorioretinal scar, a hallmark of recurrent presumed ocular toxoplasmosis. Middle row (**C**, **D**): Healed (scarred) chorioretinal lesions. **C** Patient 02, RY: a round, sharply circumscribed atrophic scar with central hyperpigmented and hyperreflective areas surrounded by a pigmented halo, located in the macular region. **D** Patient 11, LE: a round, well-demarcated, densely pigmented chorioretinal scar in the posterior pole, representing the inactive, cicatricial stage of the disease. Bottom row (**E**, **F**): Control fundi. **E** Patient 24, LE and **F** Patient 25, LE, both showing a normal retinal background, preserved vascular architecture, physiological optic disc coloration and cupping, and absence of any focal chorioretinal scarring or active inflammatory infiltrate, representing the non-toxoplasma ocular disease control group. Yellow arrows indicate the corresponding lesion in each image, as labeled (“Active focus”/“Chorioretinal scar”). Images were obtained by standard digital retinography (posterior pole field) and are displayed at comparable magnification to facilitate qualitative comparison between disease stages (active *vs*. scarred) and the control group. RY = oculus dexter; LE = oculus sinister

**Fig. 2:**
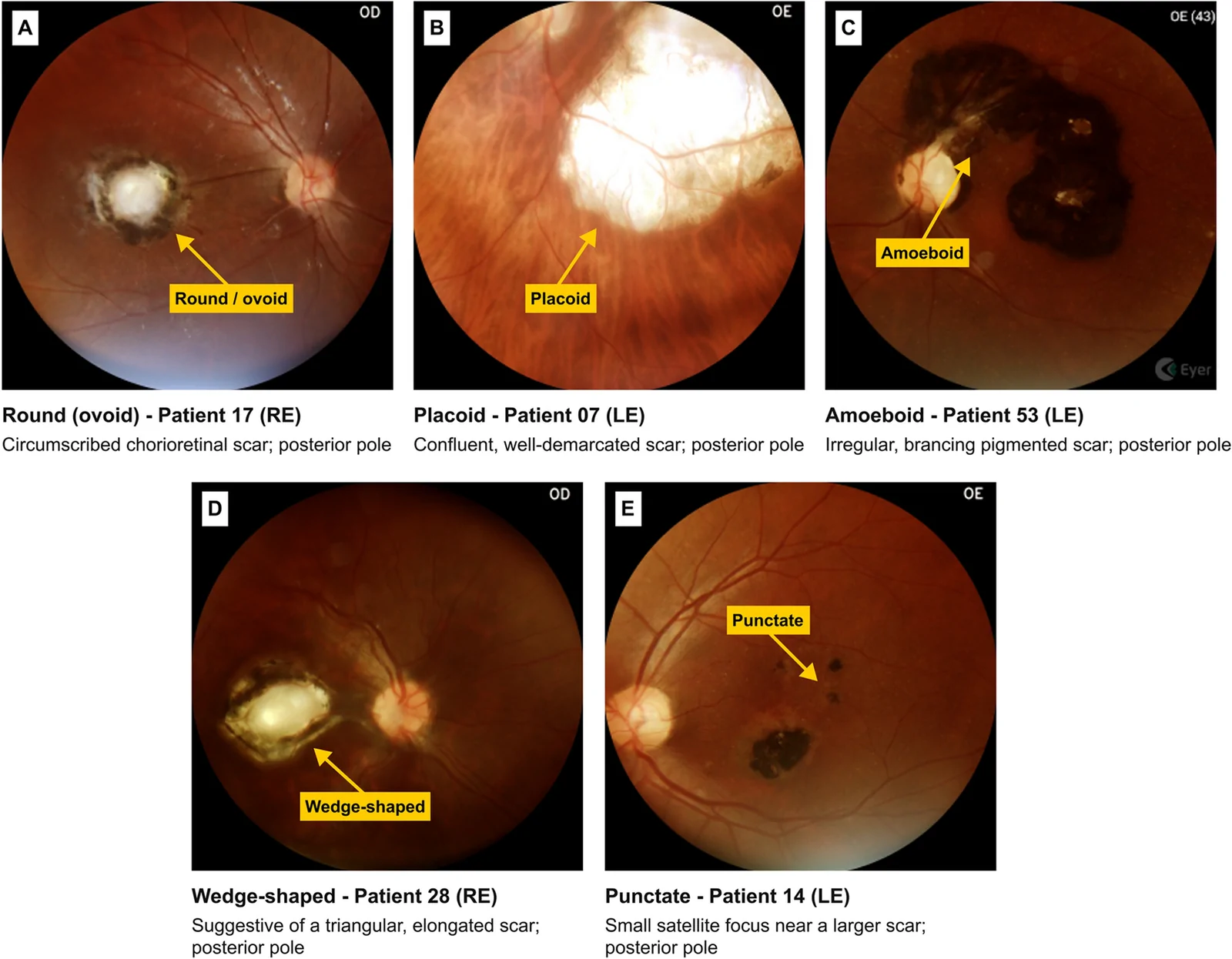
Morphological spectrum of cicatricial retinochoroidal lesions in presumed ocular toxoplasmosis, using a portable retinal camera (Eyer, Phelcom Technologies, Brazil). Representative fundus photographs illustrating the five geometric categories used to classify the morphology of cicatricial retinochoroidal lesions in the present study, defined according to the descriptive framework adapted from the Standardization of Uveitis Nomenclature (SUN) Working Group. **A** Round (ovoid)—Patient 17 RE: a healed retinochoroidal lesion with a rounded shape, pigmented borders, well delimited, with well-defined margins; the most frequent morphological pattern. **B** Placoid—Patient 07 LE: an extensive, plaque-like lesion with irregular, confluent borders, flat, with well-demarcated margins occupying an extensive area of the posterior fundus. **C** Amoeboid—Patient 53 LE: an irregular, elongated lesion with extensions and centrifugal progression resembling pseudopods. **D** Wedge-shaped—Patient 28 RE: suggestive of a triangular, elongated lesion. **E** Punctate—Patient 14 LE: multiple small pigmented spots at the level of the pigment epithelium/choriocapillaris. Yellow arrows indicate the lesion corresponding to each morphological category. All images were obtained by digital retinography using the Eyer device

Avoidance of circular classification. Group allocation (G2 versus G3) was determined exclusively on lesion activity, defined by the presence or absence of border blurring and vitreous inflammation. Lesion morphology (round or ovoid, amoeboid, placoid, wedge-shaped, punctate), anatomical location, laterality, and lesion size were not used for diagnostic or group-allocation purposes. (Figs. 1 and 2) These variables were recorded independently after group allocation and subsequently compared descriptively between G2 and G3, thereby avoiding circularity between the classification criteria and the outcomes analyzed.

#### Inclusion criteria

Individuals of both sexes, aged ≥ 15 years, presenting fundoscopic findings suggestive of retinochoroiditis presumably caused by *Toxoplasma gondii*.

#### Exclusion criteria

Individuals aged < 15 years, with clinical conditions unrelated to ocular toxoplasmosis that precluded fundoscopic evaluation; lack of cooperation for image acquisition; presence of other infectious or parasitic diseases, blood dyscrasias, coagulation disorders, or use of anticoagulants; and discordant serological results between the techniques employed.

### Statistical analysis

Inferential statistical analysis of quantitative variables was initially performed using the Kolmogorov–Smirnov test to assess data normality. As the data did not follow a normal distribution, comparisons between groups were conducted using the non-parametric Kruskal–Wallis test. Categorical variables were analyzed using Pearson’s chi-square test to compare observed frequencies between groups. A significance level of *p* ≤ 0.05 was adopted for all analyses. Statistical analyses were performed using Statistical Package for the Social Sciences (SPSS) (IBM Corp., version 23.0, 2014), GraphPad Prism (version 6.10, 2015), and GraphPad InStat (version 3.10, 2009).

Categorical variables are presented as absolute and relative frequencies with 95% confidence intervals (95% CI) calculated by the Wilson score method; denominators are stated explicitly for every proportion. Continuous variables are presented as mean, standard deviation, median and range.

### Multivariable analysis

To identify factors independently associated with lesion activity, a multivariable logistic regression model was fitted with active disease (G2 *versus* G3) as the dependent variable and age, sex, laterality and lesion size as covariates; results are reported as odds ratios (OR) with 95% CI. A complementary logistic model was fitted for bilaterality, and a linear regression model was fitted for lesion size (dependent variable) with age and sex as covariates. Given the limited sample size, the number of covariates was restricted a priori and these analyses are considered exploratory and hypothesis-generating rather than confirmatory.

### Correlation with visual function

The association between lesion size, lesion location (posterior pole *versus* mid-periphery/periphery) and best-corrected visual acuity (converted to logMAR) was assessed using Spearman rank correlation and the Mann–Whitney U test, as appropriate.

## Results

A total of 85 patients evaluated at the service were included in the study. Participants were recruited through referrals from the public healthcare system or voluntary enrollment and were subsequently classified into three groups: control, active lesions, and inactive (cicatricial) lesions of presumed toxoplasmic retinochoroiditis. Representative color fundus photographs of active and cicatricial lesions and controls are presented in Fig. 1. Of the total sample, 35 patients (41.2%) were male: 16 in the control group, 8 in the active lesion group, and 11 in the inactive lesion (scar) group, while 50 individuals (58.8%) were females, distributed as 23 in the control group, 5 with active lesion, and 22 with scar lesions.

The mean age of the study population was 45.7 ± 17.9 years, with a median of 45 years and a range from 15 to 91 years. Sixteen patients (18.8%) were older than 60 years, while 69 (81.2%) were younger than 60 years. Regarding lesion laterality, the analysis included patients from the active (G2) and cicatricial (G3) groups. Unilateral involvement was observed in 78.2% of cases, while bilateral involvement occurred in 21.8%. For morphological analysis, only eyes from these groups with retinochoroidal lesions that could be classified according to the proposed categories were included. Cases in which morphological variables were not applicable, either due to the absence of a classifiable lesion or failure to meet predefined morphological criteria, were grouped under the category “Not applicable.” Representative fundus photographs illustrating the five geometric morphological patterns identified in the study are presented in Fig. 2.

Lesion morphology, characteristics, and anatomical location are summarized in Tables 1, 2 and 3. The distribution of lesion morphology was further examined according to clinical activity. Morphological patterns were further examined according to clinical activity. Round-to-ovoid lesions predominated in both groups, occurring in 6 observations in G2 and 24 in G3, followed by amoeboid lesions (2 in G2 and 6 in G3) and placoid lesions (2 in each group). Wedge-shaped lesions were not observed in G2 and were identified in 2 observations in G3, and the same distribution was observed for punctate lesions, which were absent in G2 and identified in 2 observations in G3. Cases classified as not applicable were recorded separately (3 in G2 and 1 in G3). Regarding lesion characteristics, circumferential lesions predominated in both groups, occurring in 7 patients in G2 and 29 in G3. Confluent lesions were identified in 4 patients in G2 and 2 in G3, while 2 patients in each group were classified as not applicable. Because several morphological categories contained only a small number of observations, particularly in the active lesion group, these findings were interpreted descriptively and were not considered sufficient to establish morphology-specific differences according to lesion activity. Overall, among the 50 classifiable morphological observations, round-to-ovoid lesions were the most frequent pattern (n = 30; 60.0%), followed by amoeboid (n = 8; 16.0%), placoid (n = 4; 8.0%), wedge-shaped (n = 2; 4.0%), and punctate (n = 2; 4.0%) patterns; 4 observations (8.0%) were classified as not applicable (Table 1). Regarding lesion characteristics, 38 of 46 patients (82.6%) were classified as having circumferential lesions, whereas 6 (13.0%) were classified as having confluent lesions and 2 (4.3%) as not applicable (Table 2).

**Table 1:** Morphological characteristics of presumed ocular toxoplasmic retinochoroiditis lesions according to clinical activity in patients from Três Lagoas, Mato Grosso do Sul, Brazil

Morphology	G2 active n (%)	G3 Cicatricial n (%)
Round-ovoid	6 (46.2)	24 (64.9)
Placoid	2 (15.4)	2 (5.4)
Amoeboid	2 (15.4)	6 (16.2)
Wedge-shaped	0 (0.0)	2 (5.4)
Punctate	0 (0.0)	2 (5.4)
Not applicable	3 (23.1)	1 (2.7)
Total	13 (100)	37 (100)

N, Number of lesion observations; NA, Not applicable. Percentages were calculated within each clinical activity group

**Table 2:** Characteristics of ocular lesions caused by *Toxoplasma gondii* infection in patients from Três Lagoas, Mato Grosso do Sul, Brazil

Lesion characteristics	G2 Active n (%)	G3 Cicatricial n (%)
Circumferential	7 (53.8%)	29 (87.9%)
Confluent	4 (30.8%)	2 (6.1%)
Not applicable	2 (15.4%)	2 (6.1%)
Total	13 (100%)	33 (100%)

**Table 3:** Location of ocular lesions caused by *Toxoplasma gondii* infection in patients from Três Lagoas, Mato Grosso do Sul, Brazil

Lesion location							
Posterior pole		Med-periphery		Peripheral retina		NA	
N	%	N	%	N	%	N	%
28	52.8	10	18.9	13	24.5	2	3.8

N, Sample number; NA, Not applicable

Most patients presented lesions with a round-to-ovoid configuration (n = 30), followed by amoeboid (n = 8), placoid (n = 4), wedge-shaped (n = 2), and punctate patterns (n = 2) (Table 1). Regarding lesion characteristics, the majority were described as circumferential (n = 38; 82.6%), whereas a smaller proportion were classified as confluent (n = 6; 13.0%) (Table 2). In terms of anatomical distribution, 53 lesions in 46 patients were evaluated, with two categorized as “Not applicable.” Among the classifiable lesions, most were in the posterior pole (n = 28; 52.8%), followed by the mid-periphery (n = 10; 18.9%) and peripheral retina (n = 13; 24.5%) (Table 3).

Comparative serological analysis between groups (Table 4) included all 85 patients with available data. In the control group (G1), 15 patients had negative serology for both IgG and IgM, and 24 had positive IgG and negative IgM antibodies. In the active lesion group (G2), 10 patients were IgG positive only and three were positive in both IgM and IgG. In the cicatricial lesion group (G3), two patients were seronegative and 31 were positive just for IgG antibodies. Overall serological analysis of IgG and IgM, regardless of study group (Table 5), showed that 17 patients (20.0%) were seronegative, 65 (76.5%) were IgG positive, and three (3.5%) were IgG and IgM positive. No patients presented positivity just for IgM antibodies. All percentages were calculated based on the total sample (n = 85).

**Table 4:** Serological profile of *Toxoplasma gondii* infection in patients attended at ophthalmology clinic in Três Lagoas, Mato Grosso do Sul, Brazil

Serological profile	Control group (G1, N = 39)	Active lesion group (G2, N = 13)	Scar lesion group (G3, N = 33)	*P* value*
	N (%)	N (%)	N (%)	
IgG −/IgM-	15 (38.5%)	0	2 (6.1%)	< 0.0001
IgG +/IgM-	24 (61.5%)	10 (76.9%)	31 (93.9%)	
IgG +/IgM+	0	3 (23.1%)	0	

N, Number of patients

**Table 5:** Anti–*Toxoplasma gondii* IgG and IgM antibody profile in patients attended at ophthalmology clinic in Três Lagoas, Mato Grosso do Sul, Brazil

Serological profile	Number of patients	(%)
IgG −/IgM-	17	20.0%
IgG +/IgM-	65	76.5%
IgG +/IgM+	3	3.5%
Total	85	100.0%

Additionally, lesion size analysis was performed. Among 33 included patients, 40 eyes were affected, of which 36 were eligible for analysis. Four eyes were excluded due to inability to reliably assess lesion size: three because of extreme peripheral location, which precluded accurate measurement, and one due to absence of a retinochoroidal lesion, presenting only isolated optic neuritis. In each eligible eye, the largest retinochoroidal lesion was measured in square millimeters (mm^2^), followed by conversion to optic disc diameters, using the mean disc diameter obtained from control cases (3.0 mm^2^) as reference. The mean lesion size among the 36 analyzed eyes was 16.2 mm^2^.

## Discussion

Toxoplasmosis is a globally distributed zoonosis and remains a significant public health concern, particularly in developing countries, affecting approximately one-third of the world’s population. Although *T. gondii* infection is often asymptomatic in immunocompetent individuals, its chronic course and potential for ocular involvement result in considerable morbidity, especially in cases of retinochoroidal disease, with direct impact on visual function and patients’ quality of life [2, 21].

Given the severity and irreversibility of ocular lesions associated with toxoplasmosis, studies aimed at characterizing the clinical, serological, and morphological patterns of the disease are essential to improve diagnostic, therapeutic, and follow-up strategies. In this context, the present study provides relevant data by describing, for the first time, the profile of ocular toxoplasmosis in patients treated within the public healthcare system of Três Lagoas (MS), highlighting regional particularities that warrant attention.

The predominance of unilateral involvement observed in this cohort is consistent with previous reports, which indicate that approximately 72–86% of toxoplasmic retinochoroiditis cases affect only one eye [24]. This pattern underscores the importance of thorough ophthalmologic examination, as lesions may present as isolated, multiple, or adjacent to preexisting scars. Clinical diagnosis, based on dilated fundus examination combined with specific serological testing, remains the primary method for identifying ocular toxoplasmosis [24, 25].

Serological analysis revealed a high rate of anti–*T. gondii* IgG positivity, consistent with the known high prevalence of infection in the general population. However, the interpretation of serological markers, particularly IgM and IgA, should be approached with caution. Advances in laboratory techniques have increased the sensitivity of these assays, allowing detection of these immunoglobulins for prolonged periods after acute infection, thereby limiting their utility as exclusive markers of recent infection [26, 27]. Therefore, integration of clinical, ophthalmologic, and laboratory findings remains essential for accurate interpretation.

The additional descriptive analysis of serological status in relation to lesion activity and morphology provided further clinical context for the observed findings. IgG positivity predominated among patients with cicatricial lesions, whereas the small number of IgM-positive cases was restricted to the active lesion group. This pattern is compatible with the predominantly chronic nature of the disease and the limited specificity of isolated IgG positivity for determining the timing of ocular infection. However, because only three participants were IgM-positive, comparisons involving IgM status and individual morphological categories should be interpreted cautiously and descriptively rather than as evidence of a statistically established association. The observed distribution therefore supports the interpretation of serology as complementary evidence rather than a determinant of lesion morphology or activity.

The descriptive comparison of lesion morphology according to clinical activity showed that round or ovoid lesions predominated in both active and cicatricial disease. Amoeboid, placoid, and wedge-shaped configurations occurred less frequently, with some categories represented by only one or a few observations. The predominance of round-to-ovoid lesions across both groups suggests that this morphological configuration was not restricted to a particular stage of lesion activity in the present cohort. However, the small number of observations within several categories limits the interpretation of differences between G2 and G3, and the findings should therefore be regarded as descriptive rather than as evidence of morphology-specific determinants of lesion activity.

A higher proportion of women was observed among patients diagnosed with ocular toxoplasmosis, in agreement with previous findings [28]. Nevertheless, earlier studies suggest that clinical outcomes and visual prognosis tend to be similar between sexes, despite possible differences in presentation and lesion characteristics [29]. These variations indicate that demographic, behavioral, or regional factors may influence the observed profile, reinforcing the importance of context-specific analyses.

Regarding age distribution, most patients were younger than 60 years, consistent with studies reporting higher frequency of toxoplasmic retinochoroiditis in young adults. However, disease severity may increase with advancing age, possibly due to progressive immunological changes [30, 31]. The influence of age on epidemiology, clinical presentation, and recurrence patterns represents an important consideration in patient follow-up.

Morphological analysis of retinochoroidal scars demonstrated a predominance of unilateral lesions, with a higher concentration in the posterior pole, consistent with studies conducted in Brazil and other endemic regions [24]. Overall, the morphological patterns observed were similar to those described in the literature, except with respect to the mean size of cicatricial lesions.

The finding of a mean lesion size of 16.2 mm^2^ represents one of the main results of this study and suggests a more extensive presentation compared to previous reports [32]. This difference raises the hypothesis that regional factors may influence the severity of ocular toxoplasmosis, including potential variations in the virulence of circulating *T. gondii* strains, delays in diagnosis and treatment initiation, as well as socio-environmental factors and limited access to specialized ophthalmologic care.

When expressed in optic disc diameters, the mean lesion size corresponded to approximately 5.25 disc diameters. This conversion was based on the mean optic disc area obtained from the control group (3.0 mm^2^), considered consistent with the variability reported in the literature and with parameters adopted by the Brazilian Glaucoma Consensus [33]. It is important to note that factors such as ethnicity, refractive error, and individual anatomical variation may influence optic disc size and should be considered when interpreting these results [34].

### Benchmarking against published cohorts and methodological considerations

Direct comparison of lesion size with the literature is constrained by methodological heterogeneity. Most published series express toxoplasmic lesion size in optic disc diameters (DD) rather than in square millimetres, and frequently report the largest linear dimension rather than lesion area, which precludes a strictly equivalent comparison. In our cohort the mean area of 16.2 mm^2^ corresponded to approximately 5.25 disc areas when referenced to the mean optic disc area measured in our own controls (3.0 mm^2^).

Several sources of variation must be acknowledged when interpreting this comparison. First, measurements derived from non-mydriatic portable fundus cameras are subject to magnification error, which depends on axial length and refractive status and is not corrected by the Littmann or Bennett formulae in the present study. Second, peripheral lesions are subject to greater projection distortion than posterior pole lesions, potentially inflating apparent area. Third, the use of the optic disc as an internal scaling reference is itself affected by ethnicity, refractive error and individual anatomical variation. Fourth, cross-sectional ascertainment in a referral service may preferentially capture larger and more symptomatic lesions.

For these reasons, the observation that cicatricial lesions in this cohort appear larger than in previous reports should be regarded as a hypothesis-generating finding rather than as an established difference. It requires confirmation in prospective, multicentre studies employing standardised, magnification-corrected measurement protocols before regional differences in strain virulence or in access to care can be invoked as explanations.

Statistical analysis demonstrated significant differences among the control, active, and cicatricial groups with respect to serological profiles and lesion characteristics, reinforcing the distinction between different clinical stages of the disease. In contrast, no statistically significant differences were observed regarding sex distribution among groups, suggesting that this factor alone does not play a determining role in the clinical presentation.

## Limitations

This study has several limitations that should be considered when interpreting its findings. First, the cross-sectional design precludes any inference about causality, about the temporal sequence between infection and lesion development, and about recurrence, and it does not allow the natural history of the lesions to be followed. Second, the sample size is relatively small, particularly in the active lesion group (n = 13), which limits statistical power and renders the multivariable analyses exploratory; the confidence intervals reported should therefore be interpreted with caution. Third, participants were recruited from a tertiary referral service within the public healthcare system, so referral bias is likely and the cohort may over-represent more severe or symptomatic disease; the findings are consequently not directly generalisable to the community.

Fourth, and importantly, the diagnosis remained clinically presumed: molecular confirmation by polymerase chain reaction of intraocular fluids, and intraocular antibody production analysis (Goldmann-Witmer coefficient), were not performed, as these invasive procedures are not routinely justified in typical presentations. Consequently, misclassification of a small number of cases cannot be excluded. Fifth, lesion measurements were not corrected for ocular magnification, as discussed above. Sixth, genotyping of circulating *T. gondii* strains was not undertaken, so the hypothesis that regional strain virulence contributes to lesion size remains speculative. Finally, the absence of systematic longitudinal follow-up prevented assessment of the functional consequences of the lesions over time.

Taken together, the findings of this study contribute to a deeper understanding of ocular toxoplasmosis in a specific regional context, emphasizing the importance of early diagnosis, timely access to treatment, and continuous surveillance. The identification of larger-than-expected cicatricial lesions highlights the need for integrated approaches aligned with the One Health framework, aiming to reduce the visual impact of the disease and improve patients’ quality of life.

Although lesion morphology was descriptively summarized according to clinical activity, several morphological categories contained few observations, particularly in the active lesion group. Therefore, the available sample did not provide sufficient statistical stability for reliable morphology-specific comparisons between active and cicatricial lesions. The morphological findings should consequently be interpreted as descriptive rather than as evidence of independent associations with lesion activity.

Furthermore, vitreous inflammation was recorded qualitatively as part of the definition of active disease, but its severity was not graded using a standardized scale (e.g., NEI/SUN vitreous haze score), since this had not been incorporated into the original protocol; this precluded a quantitative comparison of inflammatory severity between groups.

## Conclusion

This study provides, for the first time, data on ocular toxoplasmosis in the municipality of Três Lagoas, MS, Brazil, enabling characterization of the clinical, morphological, and serological profile of the disease in patients treated within the public healthcare system. The findings demonstrate a high prevalence of *T. gondii* infection, with clinical patterns largely consistent with those described in the literature.

Notably, the study identified cicatricial retinochoroidal lesions with a relatively large mean lesion area compared with values reported in some previous studies. This finding raises the possibility that regional, clinical, or biological factors may contribute to variation in the severity of ocular toxoplasmosis, with potentially important clinical implications, including increased risk of visual impairment and functional impact in affected patients.

These results reinforce the importance of early diagnosis, timely access to specialized care, and continuous case surveillance in the region. Furthermore, they highlight the need for future investigations, preferably multicenter studies, exploring the role of regional, socio-environmental, and biological factors in the clinical expression of ocular toxoplasmosis, thereby contributing to improved prevention, management, and control strategies.

## Data Availability

No datasets were generated or analysed during the current study.
